# A holistic approach to managing cardio-kidney metabolic syndrome: insights and recommendations from the Italian perspective

**DOI:** 10.3389/fcvm.2025.1583702

**Published:** 2025-04-08

**Authors:** Massimo Iacoviello, Mauro Gori, Giuseppe Grandaliano, Roberto Minutolo, Dario Pitocco, Roberto Trevisan

**Affiliations:** ^1^ Department of Medical and Surgical Sciences, University of Foggia, Foggia, Italy; ^2^ Division of Cardiology, Cardiovascular Department, Papa Giovanni XXIII Hospital, Bergamo, Italy; ^3^ Department of Translational Medicine and Surgery, Università Cattolica del Sacro Cuore, Rome, Italy; ^4^ Nephrology, Dialysis and Transplantation Unit, Fondazione Policlinico Universitario Agostino Gemelli IRCCS, Rome, Italy; ^5^ Unit of Nephrology, Dept of Advanced Medical and Surgery Sciences, University of Campania “Luigi Vanvitelli”, Naples, Italy; ^6^ Endocrinology and Diabetes Unit, Azienda Ospedaliera Papa Giovanni XXIII, Bergamo, Italy; ^7^ Department of Medicine and Surgery, University of Milano Bicocca, Milan, Italy

**Keywords:** cardio-kidney metabolic syndrome, cardio-renal metabolic syndrome, cardiovascular-kidney metabolic syndrome, CKM syndrome, CRM syndrome, cardiovascular, renal and metabolic disorders, chronic kidney disease

## Abstract

Cardio-kidney metabolic (CKM) syndrome represents a complex and circular interplay of cardiovascular, renal, and metabolic dysfunctions, significantly contributing to global morbidity and mortality. This expert opinion synthesizes insights from a panel of Italian specialists in cardiology, nephrology, and diabetology, advocating for a holistic and integrated framework for CKM management. The recommendations underline the critical need for early identification and stratification of CKM stages, fostering an interdisciplinary approach that bridges specialties and ensures comprehensive care. Emphasizing innovative pathways for collaboration, including dynamic referral protocols, telemedicine, and shared decision-making, the proposed strategies aim to overcome structural and organizational barriers in healthcare. By promoting a unified approach, the framework seeks to streamline CKM care, enhance communication among specialists, and improve the coordination of services. This holistic strategy represents a pivotal step toward mitigating disease progression, improving clinical outcomes, and enhancing the quality of life for patients with CKM syndrome.

## 1 Introduction

The Cardiovascular-Kidney-Metabolic (CKM), also referred to as Cardio-Renal-Metabolic (CRM) syndrome, represents a complex and interdependent interplay between cardiovascular, renal, and metabolic systems, primarily encompassing heart failure, chronic kidney disease (CKD), and type 2 diabetes (T2D). Dysfunction in one system triggers or worsens dysfunction in others, perpetuating a self-reinforcing cycle that elevates morbidity and mortality globally (1–11). Cardiovascular disease (CVD) remains a major driver of this burden, and the global prevalence of CKM-related comorbidities is estimated at 25%–30%, highlighting its widespread burden and clinical seriousness (7, 12).

In response to the growing severity of CKM syndrome, the American Heart Association (AHA) recently proposed a position statement and a CKM classification system to stratify patients and facilitate early interventions (1, 2). This framework defines CKM syndrome as simultaneous dysfunction across CV, renal, and metabolic systems, categorizing the syndrome from early-stage dysfunction to advanced, irreversible organ damage. It emphasizes timely detection and holistic strategies to mitigate disease progression (1, 2, 8, 13).

This paper aims to address the unmet clinical needs of CKM syndrome and proposes integrated care solutions tailored to its systemic nature. Italian experts in cardiology, nephrology, and diabetology have collaborated to identify gaps in CKM management, focusing on early diagnosis, prognosis, and therapeutic approaches. The second core objective was to establish a standard and unified communication protocol among specialists, enabling a more seamless and collaborative approach to CKM care. This integrated approach seeks to improve outcomes by addressing comorbidities holistically and advancing care coordination between general practitioners (GPs), cardiologists, nephrologists, and diabetologists (1–4).

## 2 The pathophysiologic background of CKM

CKM syndrome is characterized by a complex, bidirectional interplay among cardiovascular, kidney, and metabolic systems, forming a self-perpetuating cycle of organ damage. Dysfunction in one system exacerbates pathology in the others, accelerating disease progression and increasing mortality risk (1, 2, 5–7, 13).

Chronic inflammation is a central driver, linking metabolic syndrome, CVD, and kidney impairment. Elevated levels of pro-inflammatory markers, such as high-sensitivity C-reactive protein and interleukin-6, reflect an underlying inflammatory state that contributes to endothelial dysfunction and atherosclerosis through mechanisms involving cytokine signaling, oxidative stress, and immune cell activation. These processes precipitate myocardial infarction and ischemic stroke. Inflammation also exacerbates insulin resistance, glucose dysregulation, and oxidative stress, accelerating diabetic kidney disease, heart failure (HF), and vascular stiffness, which further impair renal and cardiovascular function (1, 2, 5–7, 13, 14). Endothelial dysfunction amplifies this cycle through reduced nitric oxide availability, increased vasoconstriction, and glomerular sclerosis, resulting in proteinuria and albuminuria, markers of kidney damage that worsen cardiovascular strain. Neurohormonal Activation, driven by renin–angiotensin–aldosterone system (RAAS) and sympathetic nervous system dysregulation, contributes to vasoconstriction, sodium retention, fibrosis, and maladaptive cardiac remodeling, perpetuating systemic organ dysfunction (1, 2, 5–7, 13, 14).

The pathophysiology and progression of CKM syndrome should be best understood as an evolving continuum rather than a static or linear process. Without intervention, early metabolic disturbances and initial organ damage progress to CKD, cardiac dysfunction, and worsening insulin resistance, ultimately leading to irreversible damage in advanced stages, such as HF and end-stage kidney disease. Understanding CKM as a progressive trajectory emphasizes the need for early, system-wide interventions to disrupt this cycle before irreversible damage occurs.

## 3 Staging and risk stratification in CKM patients

In light of these considerations, the current staging of CKM proposed by the AHA should be reconsidered. While the AHA model categorized CKM syndrome into five distinct stages (stages 0–4), each of these stages has been analyzed and redefined to incorporate novel insights and perspectives. The AHA classification provides a detailed framework; however, in daily clinical practice, its multiple intermediary stages may sometimes delay the recognition of disease progression or create inconsistencies in patient management. To enhance clinical applicability, this reexamination has led to a more practical and holistic approach, consolidating these stages into two broad categories: early-stage disease and advanced disease, emphasizing proactive intervention from the earliest signs of dysfunction, and ensuring timely prevention and management while maintaining a dynamic, risk-based approach aligned with actual organ involvement

### 3.1 Early stages: from risk factors to subclinical changes

The early stages involve patients at risk or with early organ damage but without overt clinical manifestations.

“Stage 0” includes individuals with no cardiovascular, renal, or metabolic risk factors and no evidence of organ damage. At this stage, maintaining a healthy lifestyle is essential to prevent the onset of risk factors and disease progression.

“Stage 1” comprises patients presenting with early metabolic risk factors, including abdominal obesity, hypertension, or dyslipidemia. Unlike the AHA classification, which defines stage 1 solely by excess adiposity—likely reflecting the epidemiological characteristics of the US population, where obesity is a predominant concern—our approach incorporates additional metabolic risk factors. While obesity is also a significant concern in Italy, this broader perspective allows for a more comprehensive metabolic risk assessment, facilitating early identification of high-risk individuals. Recognizing these factors at an earlier stage enables timely interventions through lifestyle modifications, weight management, and metabolic control, ultimately reducing cardiovascular and renal complications.

“Stage 2” represents a later phase in which metabolic risk factors become more pronounced. Patients may exhibit impaired glucose tolerance, recently diagnosed diabetes, resistant hypertension (requiring at least two antihypertensive drugs plus a diuretic), or CKD with eGFR >45 ml/min/1.73 m^2^. Persistent albuminuria and metabolic abnormalities like hypertriglyceridemia may also appear. These patients are at high risk of progression and require intensive interventions targeting glycemic control, lipid management, anti-albuminuric strategies, and blood pressure optimization.

### 3.2 Advanced disease: from subclinical to overt organ dysfunction

Advanced stages indicate significant organ involvement and clinical manifestations.

“Stage 3” is characterized by subclinical organ dysfunction, meaning damage occurs in the absence of symptoms and involves multiple systems. Cardiovascular markers include coronary calcification, elevated BNP/NT-proBNP or hs-troponin, ejection fraction (EF) < 50%, moderate-to-severe diastolic dysfunction, and carotid atheromasia. Kidney involvement includes severe CKD (eGFR 30–45 ml/min/1.73 m^2^), rapid progression (eGFR decline of 7–8 ml/year), nephropathy diagnosis, and Urine Albumin-to-Creatinine Ratio (UACR) > 300 mg/g. In this stage of CKM syndrome, a holistic approach becomes essential to recognize and address cardiac, renal, and metabolic complications.

“Stage 4” represents the most severe phase of CKM syndrome, where patients exhibit overt clinical symptoms and advanced organ damage, including end-stage kidney disease, advanced HF, or acute cardiovascular events, including myocardial infarction or acute coronary syndrome. The complexity of these cases requires shared holistic management as an alternative to the traditional referrals where the primary specialist is selected based on the patient's predominant comorbidity.

### 3.3 The dynamic referral throughout the CKM continuum

To enhance adherence to recommendations for CKM management, it is essential to adopt a holistic approach where all clinicians are equipped to address key parameters across disciplines from early to late stages (15–17). Cardiologists, nephrologists, diabetologists and GPs must develop a shared understanding of overlapping conditions, including diabetes, CKD, and CVD. This integrated knowledge enables tailored interventions aligned with the patient's disease severity and clinical needs.

GPs play a pivotal role in early CKM identification and management (1, 2, 18). They are uniquely positioned to screen for risk factors such as hypertension, obesity, and elevated glucose levels. Routine assessments, including blood pressure, serum creatinine, UACR, and glycated hemoglobin (HbA1c), allow GPs to early detection of asymptomatic CKM indicators and timely interventions. Additionally, GPs guide high-risk individuals to appropriate lifestyle changes, emphasizing weight management, diet, and exercise. When risk factors are identified, or disease progression becomes evident, GPs are also responsible for referring patients to specialists for advanced care. These dynamic referrals typically involve a non-permanent evaluation, where specialists provide recommendations for additional tests and long-term management strategies. Specialists' expertise is essential for managing CKM's complex interplay between cardiovascular, kidney, and metabolic health. In cardiology, it is essential to evaluate target organ damage (e.g., left ventricular hypertrophy), systolic and diastolic dysfunctions, and subclinical cardiovascular changes. Furthermore, it is of utmost importance to stress the relevance of strict control of CV risk, which is worldwide suboptimal (19). In nephrology, it is important to confirm CKD diagnoses by using repeated UACR and creatinine tests over three months. Persistent morphological abnormalities, microhematuria, or proteinuria further guide nephrology evaluations. Monitoring of creatinine is mandatory to assess the impact of cardiological therapies on renal function. Managing diabetes necessitates an individualized approach to optimize care. If diabetes is diagnosed during cardiological or nephrological monitoring, a diabetological evaluation is necessary.

This holistic approach ensures a comprehensive CKM management strategy, with each specialist contributing to slowing disease progression and improving patient outcomes. Harmonizing diagnostic approaches across disciplines is critical to the early identification of high-risk CKM patients and tailored interventions.

## 4 The key diagnostic tools for the holistic approach to CKM patients

**Figure 1:**
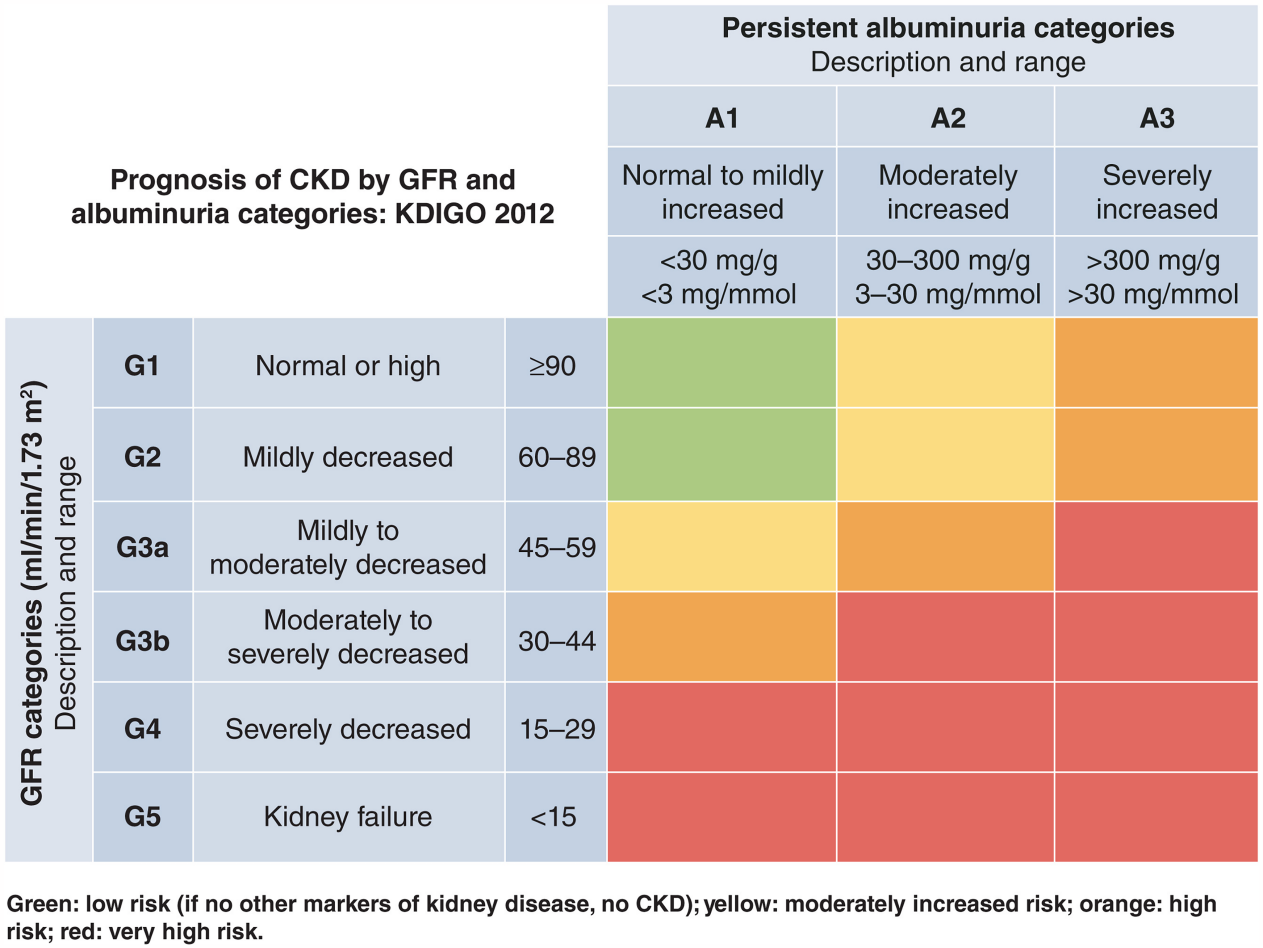
KDIGO heat map for assessing renal function and kidney damage. Source: Kidney Disease: Improving Global Outcomes (KDIGO) Diabetes Work Group. 2024 (9). Reproduced from Elsevier under a Creative Commons (CC BY-NC-ND 4.0) licence.

Several key biomarkers and diagnostic tests are critical for detecting early signs of organ damage in CKM patients, including subclinical stages. In nephrology, the Kidney Disease Improving Global Outcomes (KDIGO) classification offers a robust framework for assessing kidney function (Figure 1) (9). By combining eGFR and UACR, the KDIGO heat map stratifies patients by their global risk, including all-cause death, CKD progression and fatal and non-fatal cardiovascular events. Persistent albuminuria, even with normal eGFR, may indicate subclinical kidney damage and necessitates timely intervention. These assessments, often performed by GPs, are essential for early detection and effective risk stratification. Regular eGFR and UACR evaluations guide treatment decisions according to disease severity (20). Additionally, venous bicarbonate measurement is a valuable tool for detecting metabolic acidosis, a common CKD complication linked to muscle wasting, inflammation, and cardiovascular risk. Correcting acidosis may slow CKD progression and improve outcomes. Additionally, bioimpedance analysis provides insights into body composition, hydration, and muscle mass, aiding in nutritional interventions and optimizing care for patients at risk of sarcopenia and cardiovascular complications.

In cardiology, diagnostic tests such as ECG, natriuretic peptides (BNP/NT-proBNP), and high-sensitivity troponin (Hs-troponin) provide strong prognostic value. Elevated natriuretic peptide levels correlate with an increased risk of heart failure and help identify at-risk patients for early therapeutic intervention (21–24). Additionally, BNP/NT-proBNP evaluation should guide the echocardiogram in diabetic patients as part of the second-level screening, and both systolic and diastolic function should be evaluated, as alterations in these parameters are critical indicators of cardiovascular involvement in CKM syndrome (25). Hs-troponin is a valuable marker of myocardial injury, but its interpretation must always consider the clinical context. A single elevation in Hs-troponin does not necessarily indicate an acute coronary syndrome and should be assessed alongside serial measurements, ECG findings, and echocardiographic parameters to guide appropriate management and avoid unnecessary emergency department visits (26).

Metabolic evaluations, including blood glucose and glycated hemoglobin (HbA1c), provide vital insights into glycemic control, a key factor in slowing vascular damage and reducing cardiovascular and renal complications. Lipid profiling, blood pressure monitoring, and kidney damage assessments are also critical. In obese patients, additional tests, such as transaminases and color Doppler ultrasound of the supra-aortic trunks, further refine risk assessment.

Together, these diagnostic tests enable early detection of subclinical organ damage, risk stratification, and implementation of targeted interventions, ultimately slowing CKM progression and improving patient outcomes.

## 5 CKM stages and the role of an integrated holistic approach

The CKM staging approach facilitates identifying individuals at different levels of syndromic severity, thereby providing windows for preventive action to halt or reverse disease progression. Early detection in the preclinical phase is a primary goal, aiming to delay or avert clinical cardiovascular and kidney complications (1, 2). Active screening for CKM risk factors across the lifespan is recommended to improve prevention strategies, with screening frequency tailored to the CKM stage (1, 2).

In stages 0 and 1, the main clinical parameters include blood pressure, waist circumference, ECG, urine tests, creatinine and transaminases, in addition to blood glucose, HbA1c, and lipid profile assessments (HDL included), which collectively highlight patients at high metabolic or cardiovascular risk. GPs play a central role in identifying early abnormalities and initiating treatment, such as antihypertensives for resistant hypertension or lipid-lowering therapy for dyslipidemia. Referrals to specialists are indicated for resistant hypertension, CKD, albuminuria, newly diagnosed diabetes, or evidence of advanced disease.

Stage 2 focuses on detecting early organ stress. Primary kidney indicators include either low eGFR or increased UACR. In cases of kidney abnormalities, a kidney ultrasound is required for a comprehensive evaluation and refining diagnosis. Cardiologic assessments, including ECG, echocardiography, and BNP/NT-proBNP levels, aid in identifying subclinical cardiac damage. Monitoring HbA1c, assessing glucose intolerance, and performing Doppler ultrasound of the lower limbs are particularly relevant for diabetic patients (9). Specialists provide advanced care when required, while many patients can remain under GP supervision with periodic specialist input.

In stage 3, cardiologic findings include left ventricular hypertrophy, ejection fraction <50%, diastolic dysfunction, elevated BNP/NT-proBNP or Hs-troponin, carotid atheromas, and ECG abnormalities. Nephrologic indicators include eGFR 30–45 ml/min/1.73 m^2^ and/or UACR >300 mg/g. Patients with glomerulonephritis and/or fast progressors (eGFR reduction >7–8 ml/min per year) should remain under nephrologist care even with an eGFR >45 ml/min/1.73 m^2^. Diabetologic complications include retinopathy, microangiopathy, diabetic foot ulcers, or metabolic decompensation. At this stage, an integrated, holistic approach and close collaboration among specialists is crucial. Specialists must monitor parameters across systems, such as cardiologists evaluating kidney indicators (eGFR, UACR) and nephrologists considering cardiac biomarkers (BNP/NT-proBNP, Hs-troponin). These diagnostic tests should be repeated biannually to guide individualized care.

At stage 4, the cardiologist plays a central role, particularly for those with HF, myocardial infarction, or acute coronary syndrome. Close follow-up every 2–3 months, or more frequently during exacerbations, is critical to monitor disease progression and adjust treatment. For kidney failure patients (eGFR <15 ml/min/1.73 m^2^), the nephrologist becomes the primary case manager, supported by the cardiologist in addressing cardiovascular complications. At this stage, the nephrologist must also collaborate with the patient to decide on the most appropriate kidney replacement therapy and plan the necessary interventions, such as creating vascular access, placing a peritoneal catheter, or enrolling the patient on a waitlist for preemptive kidney transplantation. The diabetologist plays a key role in ensuring metabolic stability and glycemic control, with follow-ups approximately every 6 months.

A structured, multidisciplinary collaboration is crucial to ensure effective CKM management. While GPs play a central role in early detection and risk stratification, a shared-care model involving cardiologists, nephrologists, and diabetologists becomes essential as the disease progresses. The coordination of care should be dynamic, adapting to the predominant organ dysfunction: in early stages, GPs manage preventive strategies and referrals; in intermediate stages, specialists collaborate through periodic assessments; and in advanced CKM, the primary specialist (typically a cardiologist or nephrologist) leads the therapeutic approach.

Table 1 provides an updated summary of the main characteristics and key diagnostic tools for a holistic approach to CKM patients.

**Table 1:** Summary of updated staging; main characteristics and key diagnostic tools for a holistic approach to CKM patients.

Stage	Patient characteristics	Who is responsible	Examinations	Referral criteria
Stage 0	Individuals without cardiovascular, renal, or metabolic risk factors and no signs of organ damage	GP	Periodic monitoring of basic metabolic parameters (glucose, lipid profile, blood pressure), BMI, abdominal circumference	Not applicable (routine monitoring for primary prevention)
Stage 1	Patients with initial metabolic risk factors, including abdominal obesity, hypertension, or dyslipidemia	GP	Glucose, glycated hemoglobin, lipid profile (including HDL), creatinine, urine tests, ECG, abdominal circumference, BMI, transaminases	Nephrologist: CKD diagnosis Cardiologist: no response to therapy (2 treatment classes), abnormal ECG Diabetologist: pre-diabetes according to guidelines
Stage 2	Patients with glucose intolerance, newly diagnosed diabetes, resistant hypertension (at least 2 drugs + 1 diuretic), CKD with eGFR >45 ml/min/1.73 m^2^	GP ↔ Specialist (mainly diabetologist)	ECG, peptides, echocardiogram, renal ultrasound, supra-aortic trunks and lower limb Doppler ultrasound	Presence of characteristics of subsequent stages
Stage 3	C: Left ventricular hypertrophy, EF < 50%, moderate-to-severe diastolic dysfunction, elevated BNP, carotid atheromasia K: eGFR between 30 and 45 ml/min/1.73 m^2^, fast progressor (eGFR decline >7–8 ml/min/year), nephropathy diagnosis M: Retinopathy, microangiopathy, ulceration, inadequate glycemic control, UACR >300 mg/g	Multidisciplinary team	Clinical assessment—at least semi-annual monitoring	Holistic multidisciplinary management
Stage 4	Advanced renal-cardiovascular disease: eGFR <30 ml/min/1.73 m^2^, heart failure, AMI, ACS	Cardiologist/Nephrologist with diabetologist support	Frequent monitoring	eGFR <15 ml/min/1.73 m^2^

GP, general practitioner; AMI, acute myocardial infarction; ACS, acute coronary syndrome.

## 6 Challenges and solutions for implementing a holistic approach in the management of CKM patients in Italy

The management of CKM syndrome in Italy faces several structural and organizational barriers. A primary challenge is the limited integration of the CKM concept, encompassing cardiology, nephrology, and diabetology into clinical practice. Specialists often work in “silos”, resulting in fragmented care and inconsistent treatment strategies (1). Secondly, the lack of a shared clinical language complicates information sharing, resulting in redundant or conflicting diagnostic and therapeutic plans and ultimately affecting care quality and efficiency (27).

### 6.1 Structural and organizational barriers

Chronic patient management is frequently assigned to hospitals or specialized facilities, while territorial healthcare systems in Italy often lack centralized frameworks or a holistic approach for integrated care pathways. The healthcare system predominantly focuses on acute care, with insufficient support for chronic conditions. Limited access to advanced diagnostic tools for GPs hinders early detection and referral, fragmenting the care process. Inadequate reimbursement for essential tests, such as BNP/NT-proBNP and Hs-troponin, further constrains their use. Obesity, a key component of CKM, is often managed in private settings, creating disparities in access to care.

### 6.2 Shortage of specialists and limited referral pathways

A significant shortage of specialists, particularly nephrologists and diabetologists, exacerbates challenges in patient management, especially in less urbanized areas or regions with limited access to specialized care. Referrals between specialists are often limited to complex or acute cases, limiting opportunities for early intervention.

### 6.3 Limitations in guideline support

Current guidelines, including those provided by KDIGO, the American Diabetes Association and the European Society of Cardiology, do not adequately support a holistic approach to CKM management. They focus on risk stratification for individual conditions, such as diabetes, kidney disease, or CVD, overlooking the syndromic nature of CKM. This underscores the need for integrated guidelines addressing CKM's interconnected pathophysiology.

### 6.4 Disease-specific challenges in CKM management

#### 6.4.1 Diabetologic challenges

The shortage of diabetologists in Italy often results in suboptimal care for many diabetes patients, a core component of CKM. Furthermore, reluctance to prescribe therapies requiring frequent monitoring, such as hypoglycemic therapies, hampers effective risk evaluation, often underestimated in outpatient settings by both GPs and diabetologists.

#### 6.4.2 Cardiologic challenges

Cardiologic care for CKM patients often falls short in addressing critical risk factors such as weight and blood pressure. HF management overly focuses on left ventricular ejection fraction, neglecting diastolic function, leaving many HF patients with preserved ejection fraction unidentified. The lack of dedicated care pathways for patients with hypertension, atherosclerosis, or post-myocardial infarction results in insufficient follow-up and fragmented care. Additionally, biomarkers like Hs-troponin and BNP/NT-proBNP remain underutilized due to reimbursement limitations.

#### 6.4.3 Nephrologic challenges

Shared CKM management is hindered by the limited presence of nephrologists in territorial healthcare settings, as most nephrology care is hospital-based. There is significant confusion in the use of urinary tests; microalbuminuria is often conflated with proteinuria, and the UACR is rarely measured despite its predictive value, complicating the identification of fast progressors. Prescriptive restrictions, such as combining GLP-1 receptor agonists and SGLT2 inhibitors, limit therapeutic options for patients with declining kidney function, complicating the identification of fast progressors.

### 6.5 Proposed solutions to improve a holistic approach in the management of CKM patients in Italy

To address these challenges, the advisory board identified key strategies to improve CKM management:

#### 6.5.1 Enhancing communication and coordination

The use of telemedicine and teleconsultations was proposed as a promising solution to bridge gaps between specialists and GPs, facilitating more consistent and integrated CKM care (28, 29). Telemedicine enables real-time discussions, coordinated diagnostics, and timely adjustments in treatment plans, particularly for rural or underserved patients. Additionally, remote monitoring tools for blood pressure, glucose levels, and weight can minimize the need for frequent in-person visits, ensuring timely interventions (30, 31).

#### 6.5.2 Developing shared management models

Establishing a shared management framework between GPs and specialists can improve care continuity. GPs would handle initial screenings, risk monitoring, and preventive measures, while specialists would intervene when CKM indicators emerge (1, 2).

#### 6.5.3 Promoting a holistic culture and standardizing referral protocols and guidelines

Adopting a holistic diagnostic-therapeutic approach is essential to improving clinical outcomes in CKM patients. Specialists and GPs must understand key parameters across all CKM domains to ensure cohesive, patient-centered care. Integrated referral protocols and treatment guidelines can promote consistent management and reduce care variability (27).

#### 6.5.4 Improving access to diagnostics and therapies

Expanding reimbursement criteria for therapies and diagnostic tools, such as Hs-troponin and BNP/NT-proBNP tests, as well as simplifying the tie-consuming procedures for prescription of the newest agents, could enhance outcomes. Advocacy efforts to demonstrate their cost-effectiveness and long-term benefits are crucial.

By addressing these barriers and implementing the proposed solutions, Italy's healthcare system can advance toward a more holistic CKM approach, improving outcomes and quality of life for patients.

## 7 Therapeutic overview

In managing CKM syndrome, several pharmacological classes effectively address overlapping cardiovascular, renal, and metabolic risks. Guidelines emphasize a multidisciplinary and personalized treatment approach tailored to patients' clinical profiles (32).

Sodium–glucose co-transporter 2 (SGLT2) inhibitors are a breakthrough therapy, providing cardiovascular, renal, and metabolic benefits, including reduced HF hospitalizations, slower renal disease progression, and cardiovascular protection, even in non-diabetic patients (33–38). Their organ-protective effects make them suitable for a broad range of CKM patients. Glucagon-like peptide-1 receptor agonists, used for glycemic control, also provide cardiovascular benefits such as weight loss and reduced atherosclerotic risk (39, 40). RAAS inhibitors remain essential, offering blood pressure control, reduced proteinuria, and organ protection. Mineralocorticoid Receptor Antagonists support heart failure management but require monitoring for hyperkalemia (41). Emerging therapies, including nonsteroidal mineralocorticoid receptor antagonists, a combination of SGLT2 inhibitors with GLP-1 receptor agonists, and combined GLP-1/GIP receptor agonists, provide further options to address the multifaceted CKM features (32). Therapy should align with the CKM stage and comorbidities, ensuring efficacy and safety.

## 8 Conclusions and recommendations

Effective CKM syndrome management requires a holistic, interdisciplinary approach that integrates cardiology, nephrology, and diabetology to address the complex interplay of organ dysfunctions. While the AHA guidelines provide a useful framework, they may not fully account for variations in epidemiology and healthcare structures across different regions. A broader classification incorporating additional clinical parameters is needed to improve early risk stratification and patient management.

A structured, multidisciplinary model supported by shared protocols, telemedicine, and dynamic referral pathways can enhance coordination between specialists and primary care, improving clinical outcomes. Addressing structural and organizational healthcare system challenges is essential to ensure equitable access to early diagnosis, advanced therapies, and integrated care. A tailored, patient-centered approach that recognizes regional and systemic healthcare disparities is crucial for mitigating disease progression, improving quality of life, and reducing the global burden of CKM syndrome.

## Data Availability

All data are available from the corresponding author upon reasonable request.
